# Meath Hospital, Dublin

**Published:** 1831-01-01

**Authors:** 


### MEATH HOSPITAL, DUBLIN.

[Drs. Graves and Stokes.]

In the 5th volume of the Dublin Hospital Reports, which we have just received, and of which we shall take ample notice, we find a long and very valuable report from the above hospital, drawn up by Drs. Graves and Stokes. We have selected for this department of our journal, the following cases, reserving the others for subsequent numbers.

#### Diseases of the Arterial System.

*Case* 1. P. Magrath, aged 44, of strong habit, was admitted on the 7th Feb. 1829, with paralysis of the right lower extremity. For six months previously he had been exposed to great hardships, and early in December, 1828, became first affected with alternate sensations of heat and cold in the toes of the right foot. These sensations were soon after felt in the leg, accompanied by formication and diminution of power. Pains next supervened — and, in the course of a month, the part became cold, and totally void of sensation. The constitutional symptoms were, prostration of strength, anorexia, and constant thirst.

"On admission his intellects were perfect, and the temperature of the body, with the exception of the affected limb, natural. The pain had extended to the thigh during the night; pulse

96, small and soft. On examining the limb we found its temperature to be about 58° of Fahrenheit, and observed some œdema at the ankle and foot. There was complete loss of sensation from the middle of the thigh to the toes; the patient could rotate the thigh slightly, but no other voluntary motion of the limb was possible. The femoral artery appeared like a hard cord; it was painful on pressure, and no pulsation could be felt in the vessel. We further discovered, by the assistance of the stethoscope, that pulsation was wanting in the external and common iliac arteries on this side, while that of the left iliacs was plainly perceptible.

From these observations we came to the conclusion, that the right common and external iliacs, and the femoral artery, were in a state of permanent obstruction, which would account for the state of the limb."

The patient died three days afterwards.

*Dissection.* "No emaciation; the right lower extremity swollen and of a purple colour.

The brain, lungs, and abdominal viscera, were carefully examined, but nothing remarkable was observed, except that all these parts were usually exsanguineous. A few crude tubercles were found at the superior portions of both lungs.

The heart presented the left ventricle in the state of active aneurism, with some thickening and opacity of the aortic valves. The ascending portion and arch of the aorta were perfectly healthy, nor could any disease be discovered in the carotid or subclavian arteries, but in the innominata we observed some red patches where the lining membrane was thickened and softened. The descending aorta was healthy to within six inches from its bifurcation; here a slender red fibrinous clot was found stretching nearly to the bifurcation; beneath this clot the lining membrane was of a deep red color, thickened and soft.

The right common iliac, when viewed externally, appeared distended and livid; the left apparently healthy. On slitting down to the bifurcation, we found that the former vessel was completely plugged up from its origin by a dark clot, which extended to the external and internal iliacs, and also engaged the gluteal and obturator arteries. The same disease was found in the femoral and profunda, and extended to the origin of the anterior and posterior tibial arteries, which vessels, including the peroneal, presented a similar appearance as far as they could be traced.

Along the course of the diseased vessels, the lining membrane of the artery was found soft and thickened. It had a somewhat villous appearance, and greatly resembled an inflamed mucous membrane. In some portions the clot was separated from the vessel by a layer of dark-coloured puriform matter, in others it was adherent. The clot in the tibial arteries was not red, and much firmer than in the femoral and iliac arteries.

In the left common iliac we found the lining membrane of a deep red colour, and the vessel contained some portions of coagulable lymph. The external iliac, and femoral arteries of this side were perfectly healthy.

No disease whatever could be detected in the veins of the affected limb."

A large portion of the vasti and rectus muscles was hardened and deprived of colouring matter. The cellular tissue was œdematous.

*Case* 2. ANEURISM OF THE ABDOMINAL AORTA. The patient was a locksmith, aged 29, admitted 12th March, 1829, under the most distressing state of palpitation, of three months' standing.

"He had enjoyed good health until the beginning of November, 1828, when after exposure to cold he contracted severe pains in the loins, extending towards the umbilicus, and accompanied by a fit resembling convulsive colic. This pain of the loins presented occasional exacerbations, and was then pulsative; he referred the pulsations to the left iliac fossa.

This disease had been treated as rheumatism. Bleeding, both general and local, blistering, the use of turpentine, with other remedies, were tried, but failed to give relief. In about a month he experienced severe pain in the region of the heart, succeeded by palpitation, which has continued to the present time.

Since the commencement of this attack he uniformly experienced relief by lying on his face and right side; he could not turn on the back or left side without great pain."

His countenance was remarkably pale and exsanguious—appetite good—bowels costive—no cough.

"The impulse of the heart was greatly increased, and appeared to be communicated from an extensive surface. This impulse was still further augmented by any change of position, but particularly by turning on the back or left side, when it was felt over the anterior portion of the thorax and in the epigastrium.

Examined by auscultation, the pulsations of the heart were heard over the whole chest. During excitation we could observe a *bruit de soufflet* accompanying the action of the ventricles. Respiratory murmur every where natural."

They came to the conclusion that there was disease in some portion of the descending aorta, and that the morbid action of the heart was consequent on this. The most accurate examination, however, failed to detect any additional disease. Leeches to the loins — low diet—anodynes—occasional purgatives. In the course of a week the action of the heart was completely reduced; but was easily excited by turning on his back.

"On the eleventh day of his admission, a pulsating tumour was detected on the left side, on a level with the last dorsal vertebra. This pulsation was diffused over a space of about two square inches, which was tender to the touch; it was not accompanied by any *bruit de soufflet*, but this sound was audible in the epigastric region. He complained of pain in the tumour, increased by pressure, which also produced a feeling of nausea, and a sensation as if some fluid was ascending towards the chest.

Leeches and ice to the tumour. Use of digitalis.

The tumour gradually increased, but the pain and general distress were so much diminished that the patient left the hospital in the beginning of May. For some time his strength improved, and he lost the exsanguine appearance. He was re-admitted on the 15th of June. About three weeks previously he had attempted to resume his occupations, when he suddenly became much worse.

The tumour was not found to have increased in size, and there was no external lividity, but the pain was greatly increased, particularly at night; he then compared it to a sensation as if the bones of the pelvis were separating. The trunk was now considerably bent to the left side.

On examining the tumour a distinctly double pulsation was observable, the first stroke coinciding with the diastole of the radial artery. Two sounds were also to be heard corresponding to the pulsations of the heart, the first dull, the second clear. These phenomena bore the greatest resemblance to those produced by an hypertrophied heart.

Use of digitalis. Cold applications to the tumour.

The tumour continued to increase. He now complained of great pain during the act of passing the urine; the pain was referred to the lower part of the tumour, and subsided as soon as the act was over. This pain was severe in proportion to the distention of the bladder.

In a short time we observed that the heart was displaced; at first it was found to beat in the epigastrium, the impulse having left the usual situation. In the course of a few days it became feebler in the epigastrium, but could be felt pulsating on the right side, at the sternal end of the fifth rib, and ultimately became fixed in the intercostal space of the third and fourth ribs. Here the sound of its contractions was plainly audible, while in the natural situation they could scarcely be heard.

Soon after this occurrence he again left the hospital and indulged freely in spirituous liquors: violent action of the heart supervened, and on the following day a dreadful fit of convulsions, with pain of the abdomen and back, came on, during which he suddenly ceased to exist.

His death took place in three months from the appearance of the tumour. Dissection was not allowed."

Some judicious remarks are made on the above and on another case where there was an aneurism of the ascending aorta, which had continued five years, and where there was also the double pulsation. This double pulsation was caused, no doubt, by the "double stroke of the heart thus mechanically communicated to the sac, giving it an apparent double pulsation."

II. TRACHEOTOMY PERFORMED WITH SUCCESS FOR CHRONIC CYNANCHE LARYNGEA. By MR. PORTER.*

*Case.* Miles Brady, aged 24, a powerful looking man, admitted into the Meath Hospital, Nov. 28th, 1829, under the care of Mr. Porter.

He suffered from extreme dyspnœa, each respiration being attended with a sibilous whistling sound; the inspirations most laborious; severe exacerbations almost inducing suffocation and occurring chiefly at night. He complained of short husky cough, without expectoration; great soreness of the throat; difficulty of swallowing; and remarkable tenderness on pressing the larynx externally. As far as the fauces could be seen, there was no trace of inflammation whatever. The larynx and trachea moved rapidly upwards and downwards in the neck; the face was flushed and very red; the lips purplish; the forehead covered with profuse perspiration; the countenance inexpressibly distressed; the pulse above 100, small and weak.

As far as the history of the case could be ascertained, the patient had caught a cold about six weeks previously. The existing symptoms had appeared within the week preceding his admission, and had only been urgent during the two last days. He had previously been considered a healthy man.

* Dublin Hospital Reports. Vol. V. 8vo. pp. 628. Dublin, 1830.

"On admission he was bled to syncope, and had a bolus containing ten grains of calomel, with orders that the medicine should be repeated three times a day. At half-past 2 p. m. he was again visited, and on entering the hall of the hospital, I could distinctly hear the sound of his respiration. It is needless to repeat here the symptoms that indicate the necessity of proceeding to immediate operation; suffice it to say, that evidently he could not have endured the state of unutterable distress in which I found him for even a few hours longer, and it was almost questionable whether the lungs had not already suffered so much as to render our future efforts unavailing. After some little delay, in order to make the necessary preparations, the patient's bed was wheeled into the operation-room, and the operation performed as follows:—

Having placed the patient with his neck toward the light, and caused his head to be thrown backward as far as possible, I made an incision along the central line of the neck, commencing below near the sternum, and terminating above at the cricoid cartilage. Owing to the shortness and thickness of the neck, this incision was not of sufficient length to afford room for the further steps of the operation, a circumstance that added to its difficulty afterwards. The muscles in front of the trachea were then divided, and in the depth of the incision a vein was opened. The wound was now more than an inch deep, and from its bottom the blood bubbled up, largely mixed with air, and on applying my fingers to the neck immediately above the clavicles, the crackling sensation produced by emphysema was very evident. Having paused a few moments in order to command the hæmorrhage, I proceeded with the operation, and laid bare the trachea at a depth of more than an inch and half from the surface, and having in some degree fixed it by applying the nail of the forefinger of my left hand against the cricoid cartilage, I made a semilunar incision into it, through which a tube, formed of a portion of a gum-elastic catheter, was passed.

In this case the patient did not immediately experience the relief generally afforded by the operation: the irritation occasioned by the presence of the tube seemed to distress him greatly; he rolled himself about in the bed, looked wildly, and gnashed his teeth. The blood trickling into the trachea occasioned terrific paroxysms of coughing, in which his face became swollen and purple, and he appeared on the verge of suffocation. The tube was forcibly expelled seven or eight times, and this scene of distress continued nearly half an hour, after which he experienced some relief, became calm, and enjoyed a little sleep."

Next day, 29th, the stethoscope indicated bronchitis, and the patient was bled. Calomel continued. 30th. The bronchitis was very intense—he was harrassed with cough and difficulty of expectoration—respiration impeded by the inspissated mucus—and the tube requiring frequent removal to be cleaned. He was again bled, and calomel combined with antimonial powder, was ordered. No improvement of any consequence took place till the 3d of Dec. when ptyalism was established, and he made signs that he could swallow without difficulty—pulse 84, and soft—emphysema still extensive. The bronchitis had disappeared, according to stethoscopic indications. Venesection—calomel and opium. *Dec.* 4. The laryngeal distress continued, notwithstanding the mercurial influence on the system. On closing the wound the patient was unable to breathe or speak. He was again bled, and the blood was found to be inflamed. The other medicines to be continued. *Dec.* 13. There has been little alteration since last report. The smell from the patient's breath is abominably fetid, exactly resembling that in laryngeal phthisis. The mercury has been laid aside since the 9th December. 15*th Dec.* On attempting to drink this day, the fluids passed through the wound in the throat, "indicating a communication between the pharynx and larynx." Several symptoms of hectic fever were manifest. *Dec.* 20. A large silver canula was introduced, and seemed serviceable. The hectic symptoms increased, though the stethoscope indicated the non-existence of pulmonic inflammation. Drinks did not *entirely* pass through the wound.

"*Dec.* 22. On this day a most remarkable symptom made its appearance; the pulse had fallen to 48 strokes in the minute, but in other respects was neither feeble or irregular. On the preceding day, (Dec. 21,) he had expelled through the wound, in a fit of coughing, one of the arytenoid cartilages in a state of earthy degeneration, very much resembling carious bone. Whether this substance, which was triangular in shape, and sharp, could by its presence have kept the parts in a constant state of irritation, it is hard to determine. I have seen many instances of similar substances being discharged by cough, but the cases seemed uninfluenced by the circumstance, and went on without interruption to their fatal terminations. But here, almost immediately after its expulsion, the pulse fell to a most extraordinary degree; the capability of swallowing increased; the putrid odour became less offensive; appetite improved, and with it came some return of strength; a larger tube, the diameter of which is $\frac{3}{8}$ of an inch, was introduced with a view to its remaining permanently.

*Dec.* 28. Patient appeared wonderfully improved both in appearance and strength, although his pulse varied between 42 and 48; a very small portion of his drink passed by the wound, but the fetor had diminished, and there was no purulent expectoration: appetite very good.

*Jan.* 13. It was scarcely deemed necessary to make any report of the patient's case during the past fortnight, except that he has been gradually improving. On this day his condition is as follows:—

His strength is wonderfully recruited, but he has not recovered from the emaciation; there is a circumscribed red spot on each cheek like that in hectic fever; pulse 84. On closing the tube, he can speak in a hoarse rough tone, but he cannot respire through the natural passage. The communication between the larynx and pharynx has closed; not a drop of liquid passes through the wound. Appetite very good; sleep composed; respiration through the tube uninterrupted; no cough; no complaint of pain or uneasiness in any part: left the hospital."

On the 31st May, 1830, this man presented himself at the hospital. He was still breathing through the tube, and he will never be able to dispense with the artificial opening. The case bears a striking resemblance to that of Mr. Price, at Portsmouth, who still lives by the tube, after an interval of 15 years from the operation. In Mr. Price's case, several portions of the thyroid or arytenoid cartilages, also came away. Of this, Mr. Porter, we should have thought, was aware—and therefore we were rather surprised at his saying that he knew of no instance where sloughing of the arytenoid cartilages had occurred, and not proved fatal. He will see Mr. Price's case in the first volume of this Journal, quarterly series, for 1820-21. The case, however, is very interesting, and we return the dextrous and intelligent surgeon our thanks for a perusal of it. It reminded us of many anxious hours which we passed in attendance in a similar case.

